# Adverse impact of renin–angiotensin system blockade on the clinical course in hospitalized patients with severe COVID-19: a retrospective cohort study

**DOI:** 10.1038/s41598-020-76915-4

**Published:** 2020-11-20

**Authors:** Jeong-Hoon Lim, Jang-Hee Cho, Yena Jeon, Ji Hye Kim, Ga Young Lee, Soojee Jeon, Hee Won Noh, Yong-Hoon Lee, Jaehee Lee, Hyun-Ha Chang, Hee-Yeon Jung, Ji-Young Choi, Sun-Hee Park, Chan-Duck Kim, Yong-Lim Kim, Shin-Woo Kim

**Affiliations:** 1 Division of Nephrology, Department of Internal Medicine, School of Medicine, Kyungpook National University, Kyungpook National University Hospital, Dongdeok-ro 130, Daegu, 41944 South Korea; 2 Department of Statistics, Kyungpook National University, Daegu, South Korea; 3 Division of Pulmonology and Critical Care Medicine, Department of Internal Medicine, School of Medicine, Kyungpook National University, Kyungpook National University Hospital, Daegu, South Korea; 4 Division of Infectious Disease, Department of Internal Medicine, School of Medicine, Kyungpook National University, Kyungpook National University Hospital, Daegu, South Korea

**Keywords:** Viral infection, Risk factors

## Abstract

The association between angiotensin-converting enzyme inhibitor (ACE-I) or angiotensin II receptor blocker (ARB) and the risk of mortality in hospitalized patients with severe coronavirus disease 2019 (COVID-19) was investigated. This retrospective cohort study was performed in all hospitalized patients with COVID-19 in tertiary hospitals in Daegu, Korea. Patients were classified based on whether they received ACE-I or ARB before COVID-19 diagnosis. The analysis of the primary outcome, in-hospital mortality, was performed using the Cox proportional hazards regression model. Of 130 patients with COVID-19, 30 (23.1%) who received ACE-I or ARB exhibited an increased risk of in-hospital mortality (adjusted hazard ratio, 2.20; 95% confidence interval [CI], 1.10–4.38; *P* = 0.025). ACE-I or ARB was also associated with severe complications, such as acute respiratory distress syndrome (ARDS) (adjusted odds ratio [aOR], 2.58; 95% CI, 1.02–6.51; *P* = 0.045) and acute kidney injury (AKI) (aOR, 3.06; 95% CI, 1.15–8.15; *P* = 0.026). Among the patients with ACE-I or ARB therapy, 8 patients (26.7%) used high equivalent doses of ACE-I or ARB and they had higher in-hospital mortality and an increased risk of ARDS and AKI (all, *P* < 0.05). ACE-I or ARB therapy in patients with severe COVID-19 was associated with the occurrence of severe complications and increased in-hospital mortality. The potentially harmful effect of ACE-I or ARB therapy may be higher in patients who received high doses.

## Introduction

The outbreak of coronavirus disease 2019 (COVID-19) spread worldwide from Wuhan, China, initiating the second pandemic of the twenty-first century^1–3^. The pathogen of COVID-19 was identified as a novel betacoronavirus known as severe acute respiratory syndrome coronavirus 2 (SARS-CoV-2)^4–6^. As of July 19, SARS-CoV-2 infected more than 14 million individuals and caused 598,000 deaths worldwide^7^.

The spike protein of SARS-CoV-2 binds to the cellular receptor for intracellular entry and angiotensin-converting enzyme 2 (ACE2) is the main host protein for entry^8^. The binding of the spike protein to ACE2 results in ACE2 downregulation, prohibiting the main function of ACE2 to degrade angiotensin (Ang) II to Ang 1–7. This contributes to lung injury because the increased Ang stimulates Ang receptor 1 to enhance pulmonary vascular permeability^9,10^.

Renin–angiotensin system (RAS) blockades, such as ACE inhibitor (ACE-I) or Ang II receptor blocker (ARB), increase ACE2 expression and could enhance the entry of SARS-CoV-2 into target cells^11,12^. In contrast, ACE-I and ARB may block the excessive Ang-mediated Ang II type 1 receptor activation caused by SARS-CoV-2 and protect the infected patients against acute lung injury^13^. The role of RAS blockade in the course of COVID-19 remains controversial. In this study, we report the association between RAS blockade therapy and the risk of in-hospital mortality or severe complications such as acute respiratory distress syndrome (ARDS) and acute kidney injury (AKI) in patients with severe COVID-19 and compare the outcomes according to the doses of RAS blockade.

## Results

### Baseline characteristics

**Figure 1:**
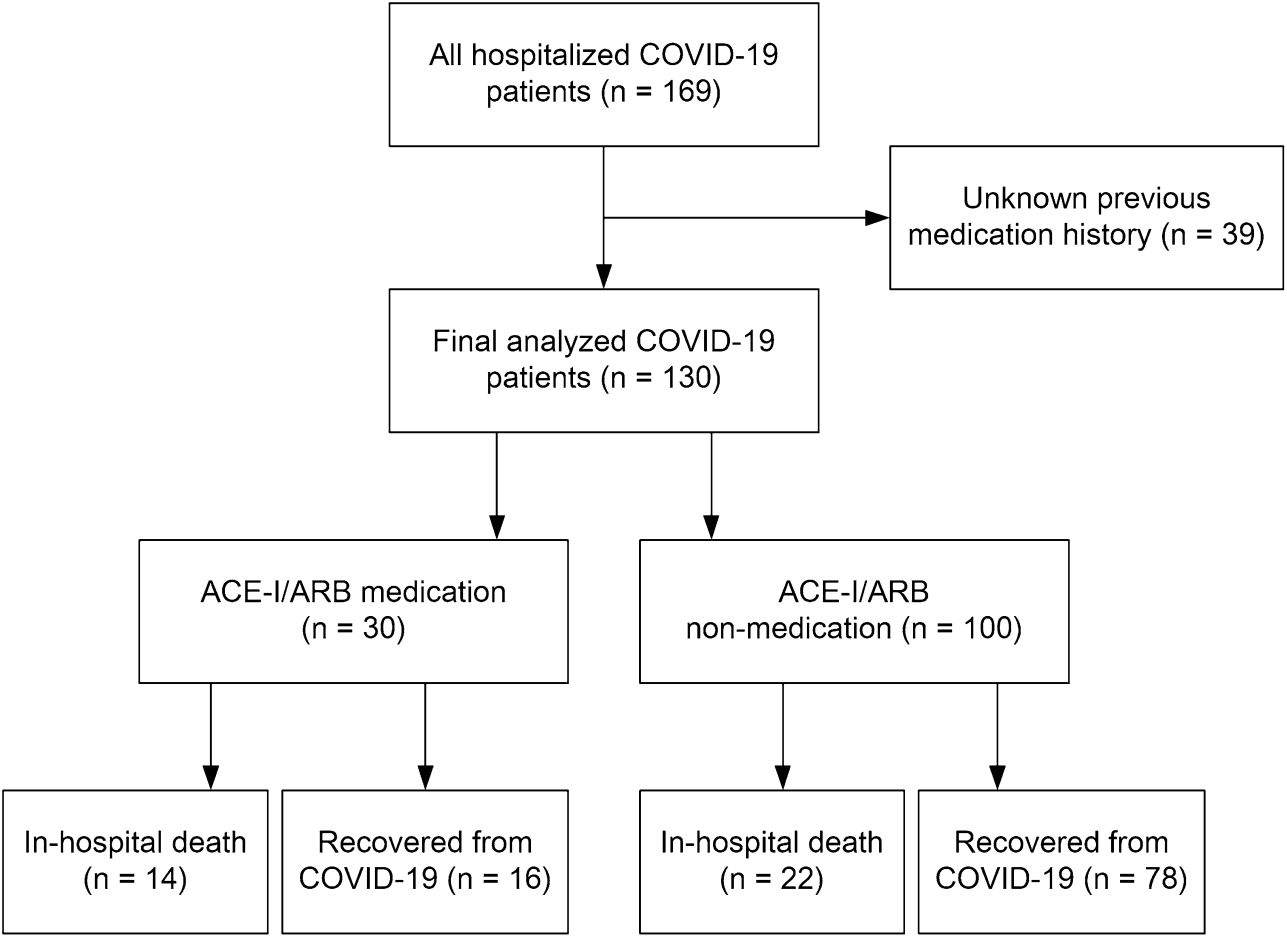
Flow diagram of the study participants.

Of the 130 hospitalized patients with severe or critical COVID-19, 30 patients (23.1%) received ACE-I (1.5%) or ARB (21.5%) therapy before hospitalization (Fig. 1). The baseline characteristics are presented in Table 1. The median age was 67.0 years and 53.8% were men. Body mass index (BMI), initial vital signs, such as blood pressure, heart rate, respiratory rate, and body temperature upon admission, and National Early Warning Score (NEWS) were not different between patients treated with and without ACE-Is or ARBs. The duration from symptom onset to COVID-19 diagnosis was also not different between the 2 groups. Patients with ACE-I or ARB medication had a higher rate of comorbid hypertension than nonmedication patients (63.3% vs 33.0%; *P* = 0.005). The rates of other comorbid diseases, including diabetes, tumor, heart diseases, and chronic kidney disease, were not different between the 2 groups (all, *P* > 0.05), and the Charlson Comorbidity Index (CCI) score was higher in patients treated with ACE-I or ARB, with borderline significance (4.1 ± 1.7 vs 3.3 ± 2.4; *P* = 0.049). Among the laboratory indices on admission, white blood cell (WBC) count and creatinine were higher among ACE-I or ARB medication patients, and estimated glomerular filtration rate (eGFR) was lower in ACE-I or ARB medication patients than nonmedication patients (all, *P* < 0.05). Other laboratory data, such as lymphocyte count, high-sensitivity C-reactive protein (hs-CRP), albumin, procalcitonin, lactate dehydrogenase, creatine phosphokinase (CPK), and ferritin, did not differ between the 2 groups (all, *P* > 0.05).

**Table 1:** Baseline characteristics.

	All (n = 130)	ACE-I/ARB (n = 30)	No ACE-I/ARB (n = 100)	*P* value
Age, years	67.0 (57.0–78.0)	72.0 (63.0–78.0)	66.0 (55.0–77.0)	0.102
Sex, male n, %	70 (53.8)	21 (70.0)	49 (49.0)	0.060
BMI, kg/m^2^ (n = 101)	23.8 ± 3.4	24.6 ± 3.8	23.5 ± 3.3	0.225
Systolic BP, mmHg	135.3 ± 24.7	142.6 ± 26.5	132.8 ± 24.0	0.060
Diastolic BP, mmHg	78.4 ± 16.0	78.1 ± 20.4	78.4 ± 14.5	0.918
Heart rate, beats per min	88.7 ± 16.8	94.0 ± 16.3	87.8 ± 17.0	0.078
Respiratory rate, breath per min	23.2 ± 12.3	26.2 ± 23.5	22.3 ± 5.8	0.380
Body temperature, ℃	37.1 ± 0.7	37.1 ± 0.6	37.1 ± 0.8	0.817
NEWS	3.9 ± 3.2	4.2 ± 3.5	3.8 ± 3.1	0.612
Days from symptom onset to diagnosis	7.8 ± 8.0	8.2 ± 7.0	7.7 ± 8.4	0.777
**Comorbid diseases, n (%)**				
Diabetes	33 (25.4)	11 (36.7)	22 (22.0)	0.150
Hypertension	52 (40.0)	19 (63.3)	33 (33.0)	0.005
Chronic lung disease	8 (6.2)	4 (13.3)	4 (4.0)	0.062
Tumor	12 (9.2)	2 (6.7)	10 (10.0)	0.580
Heart failure	3 (2.3)	1 (3.3)	2 (2.0)	0.670
Coronary heart disease	10 (7.7)	3 (10.0)	7 (7.0)	0.696
Chronic kidney disease	12 (9.2)	4 (13.3)	8 (8.0)	0.376
End-stage kidney disease	8 (6.2)	3 (10.0)	5 (5.0)	0.318
CCI	3.5 ± 2.3	4.1 ± 1.7	3.3 ± 2.4	0.049
**Laboratory findings**				
WBC count, × 10^9^/L	6.4 (4.5–8.4)	7.2 (6.1–10.7)	6.0 (4.1–7.9)	0.003
Lymphocyte count, × 10^9^/L	0.9 (0.6–1.2)	0.9 (0.6–1.5)	0.9 (0.6–1.2)	0.721
hs-CRP, mg/dL	6.4 (2.0–12.5)	6.7 (2.7–16.0)	6.1 (1.7–11.3)	0.192
Albumin, g/dL	3.5 ± 0.5	3.4 ± 0.6	3.5 ± 0.5	0.451
Procalcitonin, ng/mL (n = 82)	0.10 (0.05–0.25)	0.08 (0.03–0.57)	0.10 (0.05–0.22)	0.495
LDH, U/L (n = 111)	338.0 (233.0–494.0)	388.5 (242.0–542.5)	317.0 (232.0–469.0)	0.211
CPK, U/L (n = 85)	73.0 (49.0–177.5)	73.0 (53.0–135.0)	73.5 (45.0–211.0)	0.870
Ferritin, ng/mL (n = 91)	418.1 (243.9–843.0)	531.0 (239.0–982.0)	388.0 (245.9–781.3)	0.488
Creatinine, mg/dL	0.8 (0.7–1.3)	1.1 (0.7–1.7)	0.8 (0.6–1.2)	0.010
eGFR, mL/min/1.73 m^2^	87.0 (54.0–98.0)	62.5 (41.0–87.5)	88.0 (60.0–100.0)	0.004
**Chest radiographic findings, n (%)**				
Ground-glass opacity	56 (43.1)	15 (50.0)	41 (41.0)	0.383
Patchy consolidation	72 (55.4)	18 (60.0)	54 (54.0)	0.562

*BP*, blood pressure; *BMI*, body mass index; *CCI*, Charlson Comorbidity Index; *CPK*, creatine phosphokinase; *eGFR*, estimated glomerular filtration rate; *hs-CRP*, high-sensitivity C-reactive protein; *LDH*, lactate dehydrogenase; *NEWS*, National Early Warning Score; *WBC*, white blood cell.

### Clinical course

During hospitalization, severe complications such as ARDS and AKI have occurred more frequently in patients with ACE-I or ARB medication than in nonmedication patients (ARDS, 46.7% vs 20.0%; *P* = 0.004; AKI, 36.7% vs 14.0%; *P* = 0.006) (Table 2). The medication used to treat COVID-19 was similar between the 2 groups, and critical care rates, such as invasive mechanical ventilation, extracorporeal membrane oxygenation, and continuous renal replacement therapy, were also not different. The mean duration of hospital stay was 23.8 days, and 36 deaths (27.7%) occurred during hospitalization. Patients who survived all recovered from COVID-19. The in-hospital mortality was significantly higher in the ACE-I or ARB medication patients than in the nonmedication patients (46.7% vs 22.0%; *P* = 0.008).

**Table 2:** Comparison of complications, treatment, and clinical outcomes.

	All (n = 130)	ACE-I/ARB (n = 30)	No ACE-I/ARB (n = 100)	*P* value
Length of hospital stay, days	23.8 ± 16.5	20.3 ± 14.3	24.9 ± 17.1	0.189
**Treatment, n (%)**				
Antibiotics	99 (76.2)	26 (86.7)	73 (73.0)	0.123
Lopinavir/ritonavir	73 (56.2)	20 (66.7)	53 (53.0)	0.186
Darunavir/cobicistat	33 (25.4)	8 (26.7)	25 (25.0)	0.854
Hydroxychloroquine	91 (70.0)	24 (80.0)	67 (67.0)	0.173
Glucocorticoid	48 (36.9)	13 (44.8)	35 (35.0)	0.374
IVIG	13 (10.0)	2 (6.7)	11 (11.0)	0.731
Oxygen therapy	87 (66.9)	21 (70.0)	66 (66.0)	0.683
Invasive MV	25 (19.2)	7 (23.3)	18.0 (18.0)	0.516
ECMO	4 (3.1)	2 (6.7)	2 (2.0)	0.231
CRRT	9 (6.9)	4 (13.3)	5 (5.0)	0.210
ICU admission	38 (29.2)	10 (33.3)	28 (28.0)	0.595
**Complications, n (%)**				
ARDS	34 (26.2)	14 (46.7)	20 (20.0)	0.004
Acute kidney injury	25 (19.2)	11 (36.7)	14 (14.0)	0.006
Shock	54 (41.5)	15 (50.0)	39 (39.0)	0.284
**Clinical outcomes, n (%)**				0.008
In-hospital death	36 (27.7)	14 (46.7)	22 (22.0)	
Recovery	94 (72.3)	16 (53.3)	78 (78.0)	

*ARDS*, acute respiratory distress syndrome; *CRRT*, continuous renal replacement therapy; *ECMO*, extracorporeal membrane oxygenation; *ICU*, intensive care unit; *IVIG*, intravenous immunoglobulin; *MV*, mechanical ventilation.

### Association between ACE-I or ARB use and in-hospital mortality

**Figure 2:**
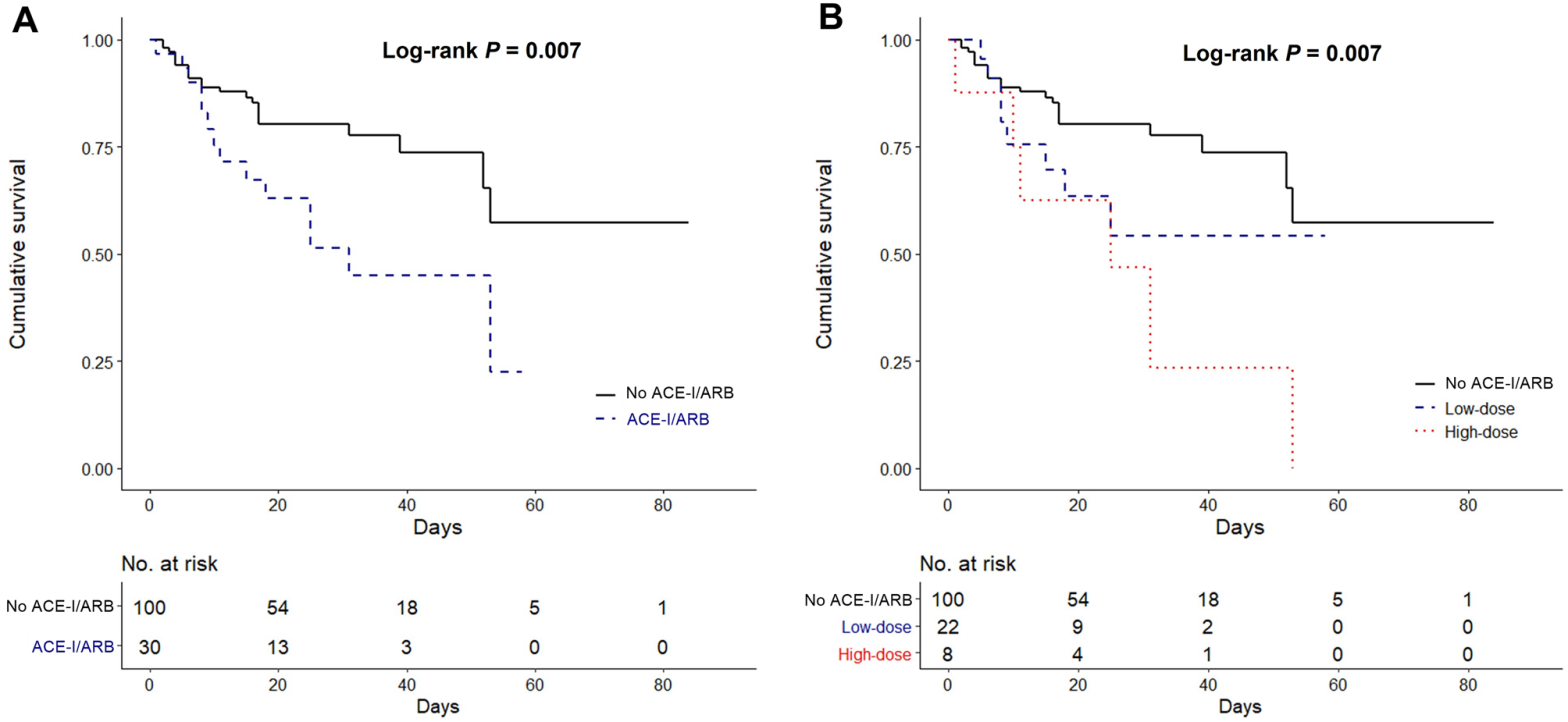
Kaplan–Meier survival curves for in-hospital mortality of patients with COVID-19. **(A)** ACE-I or ARB therapy on admission. **(B)** Dose of ACE-I or ARB on admission.

In-hospital mortality according to the ACE-I or ARB therapy before admission is shown in Fig. 2. The mortality rate was significantly higher in the ACE-I or ARB medication patients than in the nonmedication patients (*P* = 0.007; Fig. 2A). We performed the multivariate Cox regression analysis to adjust for confounding effects of variables to clearly identify the association. ACE-I or ARB therapy had significant associations with in-hospital mortality after adjusting for age (model 1: adjusted hazard ratio [aHR], 2.48; 95% confidence interval [CI], 1.26–4.88; *P* = 0.009). The higher mortality rate in the ACE-I or ARB therapy group remained significant after adjusting for age and CCI (model 2: aHR, 2.33; 95% CI, 1.18–4.60; *P* = 0.015) and for age, CCI, and WBC count (model 3: aHR, 2.20; 95% CI, 1.10–4.38; *P* = 0.025) (Table 3).

**Table 3:** Associated factors of in-hospital mortality in the Cox proportional hazard model.

Variables	Univariate		Model 1^†^		Model 2^‡^		Model 3^§^	
	HR (95% CI)	*P* value	aHR (95% CI)	*P* value	aHR (95% CI)	*P* value	aHR (95% CI)	*P* value
ACE-I/ARB medication	2.40 (1.23–4.71)	0.010	2.48 (1.26–4.88)	0.009	2.33 (1.18–4.60)	0.015	2.20 (1.10–4.38)	0.025
Age	1.77 (1.32–2.37)	< 0.001	1.83 (1.35–2.50)	< 0.001	1.61 (1.17–2.20)	0.003	1.57 (1.14–2.17)	0.006
CCI	1.28 (1.14–1.44)	< 0.001			1.23 (1.04–1.45)	0.016	1.24 (1.05–1.47)	0.013
WBC count	1.08 (1.00–1.18)	0.064					1.05 (0.96–1.15)	0.315
Sex (ref: female)	1.10 (0.56–2.14)	0.784						
Hypertension	1.71 (0.88–3.29)	0.111						

^†^Model 1: adjusted for age; ^‡^model 2: adjusted for model 1 plus CCI; ^§^model 3: adjusted for model 2 plus WBC count.

*aHR*, adjusted hazard ratio; *CI*, confidence interval; *CCI*, Charlson Comorbidity Index; *WBC*, white blood cell.

High-dose group showed higher mortality (*P* = 0.007) in the dose–effect analysis by ACE-I or ARB dose (Fig. 2B). In the multivariate Cox regression analysis to evaluate the dose effect of RAS blockade, high-dose ACE-I or ARB therapy was independently associated with increased in-hospital mortality in all models (model 1 [adjusted for age], aHR, 3.28; 95% CI, 1.32–8.15; *P* = 0.010; model 2 [adjusted for age and CCI], aHR, 3.25; 95% CI, 1.30–8.10; *P* = 0.011; model 3 [adjusted for age, CCI, and WBC count], aHR, 3.51; 95% CI, 1.39–8.88; *P* = 0.008) (Supplementary Table 1).

**Figure 3:**
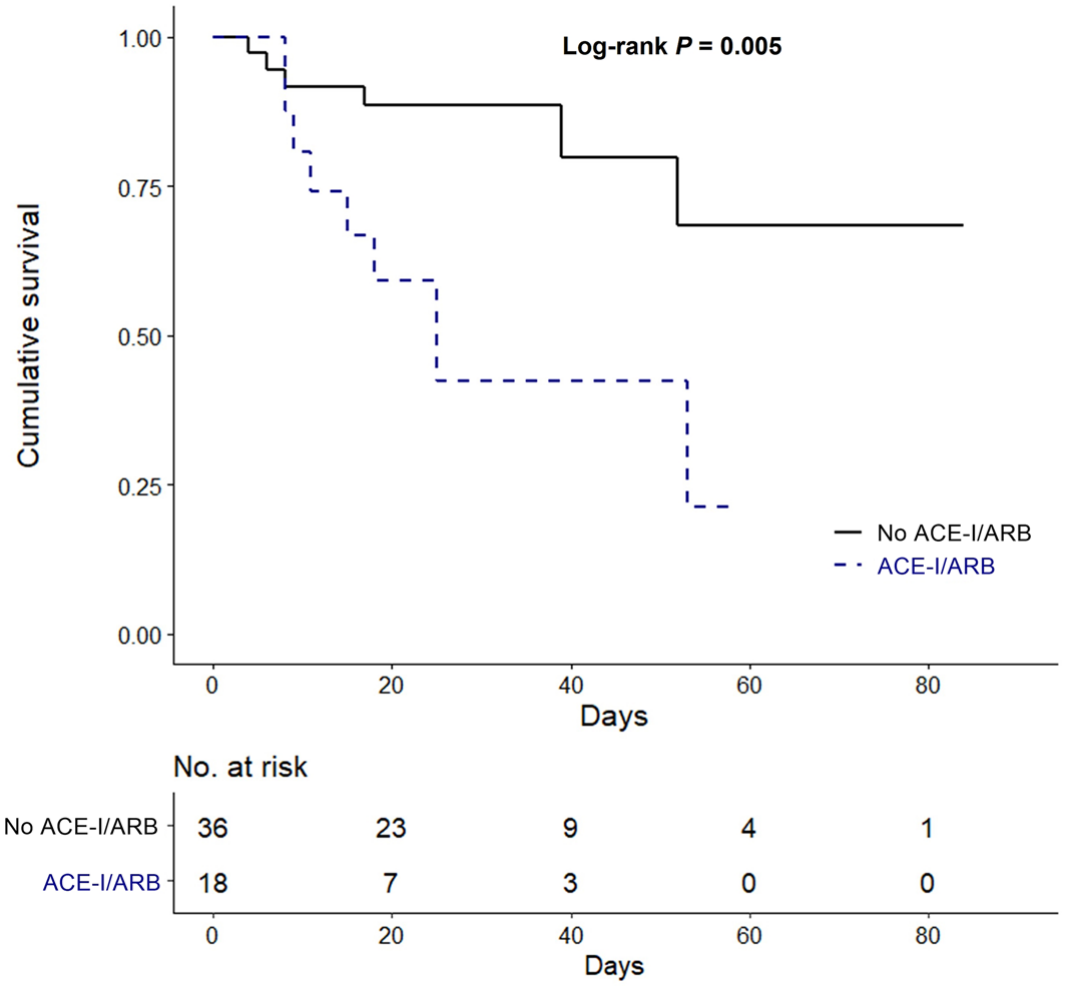
Kaplan–Meier survival curve for in-hospital mortality of patients with COVID-19 according to ACE-I or ARB therapy in propensity score matched population.

Furthermore, we analyzed in-hospital mortality using propensity score matching to compensate for the effects of confounding factors including age and comorbidities. Baseline characteristics for propensity matched population are presented in Table 4. Two groups had comparable baseline characteristics such as age, sex, BMI, NEWS, and comorbid diseases. In the Kaplan–Meier survival curve, in-hospital mortality of ACE-I or ARB medication patients was significantly higher than that of nonmedication patients (*P* = 0.005) (Fig. 3).

**Table 4:** Baseline characteristics in propensity score matched population.

	ACE-I/ARB (n = 18)	No ACE-I/ARB (n = 36)	*P* value
Age, years	68.0 (60.0–76.0)	71.0 (58.0–83.0)	0.680
Sex, male n, %	14 (77.8)	18 (50.0)	0.078
BMI, kg/m^2^ (n = 42)	25.2 ± 2.7	23.2 ± 3.6	0.116
NEWS	4.0 ± 3.5	3.9 ± 3.1	0.883
Days from symptom onset to diagnosis	8.7 ± 7.6	6.7 ± 5.8	0.317
**Comorbid diseases, n (%)**			
Diabetes	5 (27.8)	12 (33.3)	0.763
Hypertension	9 (50.0)	19 (52.8)	1.000
Chronic lung disease	2 (11.1)	2 (5.6)	0.462
Tumor	2 (11.1)	3 (8.3)	0.740
Heart failure	0 (0.0)	1 (2.8)	0.475
Coronary heart disease	1 (5.6)	3 (8.3)	0.713
Chronic kidney disease	2 (11.1)	6 (16.7)	0.588
End-stage kidney disease	1 (5.6)	3 (8.3)	0.713
CCI	3.7 ± 1.8	4.1 ± 2.5	0.586

*BMI*, body mass index; *CCI*, Charlson Comorbidity Index; *NEWS*, National Early Warning Score.

### Association between ACE-I or ARB use and severe complications

In addition to in-hospital mortality, the associations between ACE-I or ARB therapy and the occurrence of severe complications, such as ARDS and AKI, were also evaluated. The result of multivariate logistic regression analysis for severe complications is presented in Table 5. After adjusting for confounding factors that were included in the multivariate Cox regression model (age, CCI, and WBC count), ACE-I or ARB therapy was found to have a significant association with ARDS (adjusted odds ratio [aOR], 2.58; 95% CI, 1.02–6.51; *P* = 0.045) and with AKI (aOR, 3.06; 95% CI, 1.15–8.15; *P* = 0.026). Stratifying ACE-I or ARB group according to dosage, high-dose medication group had a significant association with both ARDS (aOR, 6.80; 95% CI, 1.51–30.70; *P* = 0.013) and AKI (aOR, 2.60; 95% CI, 1.08–6.28; *P* = 0.034) (Supplementary Table 2).

**Table 5:** Associated factors for severe complications in the multivariate logistic regression analysis.

Variables	ARDS		AKI	
	Adjusted OR (95% CI)	*P* value	Adjusted OR (95% CI)	*P* value
ACE-I/ARB medication	2.58 (1.02–6.51)	0.045	3.06 (1.15–8.15)	0.026
Age	1.21 (0.83–1.75)	0.320	1.08 (0.73–1.58)	0.705
CCI	1.09 (0.87–1.37)	0.458	1.15 (0.91–1.45)	0.253
WBC count	1.14 (1.01–1.29)	0.039	1.03 (0.90–1.18)	0.652

*ARDS*, acute respiratory distress syndrome; *AKI*, acute kidney injury; *CI*, confidence interval; *CCI*, Charlson Comorbidity Index; *OR*, odds ratio; *WBC*, white blood cell.

## Discussion

The effect of ACE-I or ARB therapy in hospitalized patients with severe COVID-19 was investigated in this study. Our cohort revealed comparable comorbidities between patients with and without ACE-I or ARB, except hypertension. ACE-I or ARB treatment was associated with in-hospital complications and mortality in patients with severe COVID-19 after adjusting for confounding factors and propensity score matching. In addition, the patients with a higher dose of ACE-I or ARB exhibited higher mortality than the patients without ACE-I or ARB, whereas a lower dose of ACE-I or ARB was not associated with increased mortality. This suggests that patients with COVID-19 on ACE-I or ARB therapy require more careful monitoring and intensive treatment.

Several large studies have been published to demonstrate the effect of ACE-I and ARB on the mortality of patients with COVID-19^14–16^. They reported that ACE-I or ARB treatment was not associated with mortality in patients with COVID-19. However, the conclusion regarding the use of RAS blockade in COVID-19 is still inconsistent, even among several meta-analyses. Most meta-analyses have reported that ACE-I and ARB use was not associated with mortality^17–19^, but one showed a relationship between ACE-Is and ARBs and lower mortality among hypertensive patients with COVID-19^17^. Another meta-analysis reported an overall protective effect of RAS blockade with death and critical disease^20^. The heterogeneous conclusions might be related to the fact that some population-based studies could not assess confounding factors such as obesity, severity of diabetes, and control of hypertension. Adjusting for several crucial confounding variables for the outcome of COVID-19 might result in a different conclusion.

We reported a negative impact of ACE-I and ARB use among hospitalized Korean patients. Liabeuf et al. described the association of RAS blockade use with a higher risk of severe COVID-19 in 268 hospitalized patients, which is consistent with our results^21^. The difference of the study compared with the previous reports is that all confounding factors such as BMI, blood pressure, and various laboratory data were identified in severe hospitalized patients with COVID-19. The follow-up duration for definite treatment outcomes in hospitalized patients could also make the effect of RAS blockade different. Some research was biased toward including more patients who died early in their hospital course^22^. In such cases, comparisons of long-term prognosis might yield different results. Taken together, our study suggests that the effect of RAS blockade might differ in more severe hospitalized patients with COVID-19 when all variables and treatment outcomes are considered.

The present results are similar to those reported in a nationwide population study in Korea^23^. The mortality rate of hospitalized cases was higher among RAS blockade users than nonusers, although the use of RAS blockade was not an independent risk factor in multivariate analysis. In addition, RAS blockade was independently associated with severe disease, such as the need for high-flow nasal cannula among 1954 hospitalized patients. The conflicting effect of RAS blockade compared with other studies might also be attributable to ethnic differences in ACE2 expression. Considering that the East Asian population expresses higher ACE2 in tissues than other populations^24^, ACE2 upregulation induced by RAS blockade might be more prominent in influencing the prognosis of Asian patients with COVID-19. This hypothesis should be confirmed by further prospective cohort studies or randomized controlled trials.

Severe COVID-19 was associated with multiple organ injuries such as ARDS^25–27^ and AKI^28–30^, which were identified as independent risk factors for mortality in patients with COVID-19. However, the association of ACE-I or ARB use with AKI or ARDS is still not fully understood. Some studies did not find any relationship between the use of ACE-I or ARB and the development of AKI^31,32^. Contrary to these results, Oussalah et al. reported a harmful effect of long-term ACE-I or ARB use on the renal function and further interaction with the occurrence of AKI and ARDS in 149 patients with severe COVID-19^33^. Our study highlighted the possible association between ACE-I or ARB use and a significant increase in both ARDS and AKI. The induction of ACE2 expression by RAS blockade might affect the binding of SARS-CoV-2 to kidney tissues, deteriorating renal function, considering that ACE2 is abundantly expressed in the proximal tubules of the kidney and type II alveolar epithelium of lung^34,35^.

Little is known about the dose effect of RAS blockade in COVID-19. Some studies analyzed the tolerability and efficacy of RAS blockade in high- and low-dose groups^36,37^. In the same manner, we analyzed the association of different doses of ACE-I and ARB with outcomes of COVID-19. High-dose ACE-I/ARB medication patients exhibited a higher risk of either mortality or organ damages such as ARDS and AKI. Our results suggest that ACE-I and ARB may negatively affect COVID-19, especially those who have been taking high doses. The dosing effect of RAS blockade should be investigated through the further analysis and research in patients with COVID-19.

There are several limitations that should be considered in the interpretation of our results in this study. Although our study was conducted in 2 independent hospitals, it had a retrospective design and a limited number of patients. To prove the causal relationship, our results should be investigated in larger studies with long-term follow-up. Nevertheless, the advantage of our study is that the detailed course of treatment and laboratory findings and patients’ characteristics were evaluated to confirm the association between ACE-I or ARB and prognosis. Because most of the patients were discharged and there were no critically ill patients at the end of the survey, it can be said that the investigation of outcomes was clearly evaluated in our cohort.

In conclusion, ACE-I or ARB therapy in patients with severe COVID-19 was associated with the occurrence of severe complications and increased in-hospital mortality. The effects were significant when high doses of ACE-I or ARB were administered to the patients. Our findings provide data for a harmful effect of RAS blockade on COVID-19. Further prospective trials are warranted on this class of drugs in the management of patients with COVID-19.

## Methods

### Patient population

This was a retrospective cohort study that analyzed all patients with COVID-19 who were admitted to two university-based tertiary hospitals in Daegu, South Korea (Kyungpook National University Hospital and Kyungpook National University Chilgok Hospital). COVID-19 was confirmed based on nasopharyngeal and oropharyngeal swab samples using real-time reverse transcriptase polymerase chain reaction for SARS-CoV-2^28^. At the time of initial diagnosis, patients with COVID-19 were classified into four categories (mild, moderate, severe, and critical) using the Telephone Severity Scoring System according to age, symptoms, underlying diseases, and social factors^38^. Briefly, patients with severe illness were suspected to have severe pneumonia with cough and fever of ≥ 38 °C. If critical patients experienced shortness of breath for more than 1 day and had a respiratory rate of 30 or more per minute, they were suspected to have critical pneumonia. Owing to the acute hospital bed shortage, only severe and critically ill patients were admitted to tertiary hospitals in Daegu, South Korea. Patient data were collected from February 17 to May 31, 2020. Among the 169 patients with COVID-19 who were admitted to the 2 hospitals, 130 patients were able to identify previous medication and were included in the analysis.

### Data collection

Confirmed patients with COVID-19 were retrieved using the ICD-10 code U071 (COVID-19, virus identified). Notably, four reviewers (J.H.K., G.Y.L, S.J., and H.W.N.) collected the data by a manual review of electronic medical records and entered the data in the predefined secure online database, and two authors (J.H.L and J.H.C.) cross-checked the entered data. Baseline patient information, including demographics, comorbid diseases, symptoms, medications before hospital admission, and vital signs, and data of in-hospital course, including treatment information, complications such as ARDS, AKI, shock, and deaths, discharge date, and length of hospital stay were collected. Oral medication history and dose, including ACE-Is and ARBs, were extracted retrospectively. The occurrence of complications and the date were investigated retrospectively by reviewers according to the definition. The laboratory dataset on admission included WBC count (10^9^/L), lymphocyte count (10^9^/L), hs-CRP (mg/dL), albumin (g/dL), procalcitonin (ng/mL), lactate dehydrogenase (U/L), CPK (U/L), ferritin (ng/mL), and creatinine (mg/dL) were also individually investigated from the electronic medical records. The eGFR (mL/min/1.73 m^2^) was calculated using the Chronic Kidney Disease Epidemiology Collaboration Equation^39^. All collected data were validated by manual verification. The study protocol was reviewed and approved by the institutional review boards (IRBs) of Kyungpook National University Hospital (2020-03-044) and Kyungpook National University Chilgok Hospital (2020-04-013). Because the study did not infringe on patient’s privacy or health, both IRBs approved waiver of informed consent. This study was performed according to the Declaration of Helsinki.

### Outcome

The primary outcome was in-hospital mortality associated with COVID-19 infection. Secondary outcomes included the development of ARDS and AKI after the diagnosis of COVID-19.

### Definition

Shock was defined as a systolic blood pressure of < 90 mmHg for > 30 min or requiring the use of vasopressors to maintain a systolic blood pressure of > 90 mmHg^40^. According to the Kidney Disease Improving Global Guidelines, AKI was defined as (1) an increase in serum creatinine of 0.3 mg/dL or more within 48 h, (2) an increase in serum creatinine of 1.5 or more times than the baseline, or (3) urine volume of < 0.5 mL/kg per hour for 6 hours^41^. ARDS was defined according to the Berlin Definition^42^. The NEWS, which is an early warning score facilitating the early recognition and response to patient deterioration^43^, consists of seven parameters: respiratory rate, peripheral oxygen saturation, use of supplemental oxygen, body temperature, systolic blood pressure, heart rate, and neurological status. Each parameter is assigned a score of 0 to 3. The CCI score was developed as a prognostic classification and weighting method that predicts mortality based on patient age and comorbid diseases^44^. Regardless of medication during hospitalization, patients with ACE-I or ARB therapy were defined as those using ACE-I or ARB at the time of admission. According to the used dosage of ACE-I or ARB, ACE-I or ARB medication patients were stratified into two groups, as follows: (1) high dose (total daily dose of > 160 mg of valsartan or > 10 mg of enalapril or equivalent doses of other ACE-Is or ARBs) and (2) low dose (total daily dose of ≤ 160 mg of valsartan or ≤ 10 mg of enalapril or equivalent doses of other ACE-Is or ARBs)^36^.

### Clinical management

All patients received symptomatic care with antipyretic and antitussive agents. Hospitalized patients with COVID-19 were treated with lopinavir and ritonavir or darunavir and cobicistat with or without hydroxychloroquine. Critical patients with COVID-19 were also treated with corticosteroid or intravenous immunoglobulin per physicians’ decision.

### Statistical analysis

The normal distribution of variables was analyzed using the Kolmogorov–Smirnov test. Data are expressed as the mean ± standard deviation or median (interquartile range) based on the distribution of the variables for continuous variables and numbers (percentage) for categorical variables. The Student’s *t* test and Mann–Whitney *U* test were used for continuous variables, and the Pearson chi-square test or Fisher’s exact test was used for categorical variables, as appropriate. Kaplan–Meier analysis with log-rank test was used to compare the in-hospital mortality. Multivariate Cox regression models were performed to identify independent associations between ACE-I or ARB therapy and the primary outcome of in-hospital mortality. Variables identified as risk factors for mortality in COVID-19 were analyzed in the univariate model^45^. Variables with *P* ≤ 0.10 in univariate analyses were entered into the multivariate models. In consideration of the number of deaths to reduce the possibility of overfitting, we have limited the maximum number of variables to 4. Model 1 included demographic data (age), model 2 additionally included comorbidities (CCI), and model 3 additionally included biologic marker (WBC count). The results were presented as HRs with 95% CIs. Violation of the proportional hazards assumption was tested by means of inspection of log minus log plots. In addition, in-hospital mortality was analyzed among groups classified by ACE-I or ARB doses to evaluate the dose effect. For more accurate analysis of in-hospital mortality between groups, we used propensity score matched patient groups to balance the baseline characteristics (1:2 match). Propensity scores were calculated from a logistic regression model, using age and comorbidities, such as hypertension, diabetes, and chronic lung disease. Logistic regression models were used to analyze the secondary outcomes. Multivariate logistic regression analyses were performed after adjusting possible confounding factors that were included in the Cox proportional hazards model for mortality to determine the independent association of ACE-I or ARB therapy on severe complications, such as ARDS and AKI (model 3). SPSS version 22.0 (IBM Corp., Armonk, NY) was used for statistical analyses. *P* < 0.05 was considered statistically significant.

## Supplementary information

Supplementary Information.

## Data Availability

The datasets generated and/or analyzed during this study are available from the corresponding author, S.W.K., on reasonable request.
