# Supplementary material for: Adverse impact of renin–angiotensin system blockade on the clinical course in hospitalized patients with severe COVID-19: a retrospective cohort study
Source: Sci Rep. 2020 Nov 20;10:20250. doi: 10.1038/s41598-020-76915-4 (PMC7680105; doi:10.1038/s41598-020-76915-4)
Supplement: Supplementary file 1 — Supplementary Information. [file 41598_2020_76915_MOESM1_ESM.pdf]

Adverse impact of renin-angiotensin system blockade on the clinical course in hospitalized patients with severe COVID-19: a retrospective cohort study

Jeong-Hoon Lim[1,¶], Jang-Hee Cho[1,¶], Yena Jeon[2], Ji Hye Kim[1], Ga Young Lee[1], Soojee Jeon[1], Hee Won Noh[1], Yong-Hoon Lee[3], Jaehee Lee[3], Hyun-Ha Chang[4], Hee-Yeon Jung[1], Ji-Young Choi[1], Sun-Hee Park[1], Chan-Duck Kim[1], Yong-Lim Kim[1], and Shin-Woo Kim[1,*]

[1]Division of Nephrology, Department of Internal Medicine, Kyungpook National University School of Medicine, Daegu, South Korea

[2]Department of Statistics, Kyungpook National University, Daegu, South Korea

[3]Division of Pulmonology, Department of Internal Medicine, Kyungpook National University School of Medicine, Daegu, South Korea

[4]Division of Infectious Disease, Department of Internal Medicine, Kyungpook National University School of Medicine, Daegu, South Korea

Supplementary Information Listing:

Supplementary Table S1, and Table S2

[*]Corresponding author:

Shin-Woo Kim, M.D, PhD

Professor

Division of Infectious Disease, Department of Internal Medicine,

Kyungpook National University School of Medicine,

Dongdeok-ro 130, Daegu, 41944, South Korea

Tel: +82-53-200-6525

Fax: +82-53-426-2046

E-mail: ksw2kms@knu.ac.kr

Supplementary Table S1. Associated factors of in-hospital mortality in the Cox proportional hazard model classified by doses of ACE-I or ARB

Variables	Model 1†		Model 2‡		Model 3§	
	aHR (95% CI)	*P* value	aHR (95% CI)	*P* value	aHR (95% CI)	*P* value
ACE-I/ARB medication						
None	Reference		Reference		Reference	
Low-dose	2.09 (0.92–4.73)	0.078	1.92 (0.85–4.36)	0.119	1.67 (0.72–3.88)	0.234
High-dose	3.28 (1.32–8.15)	0.010	3.25 (1.30–8.10)	0.011	3.51 (1.39–8.88)	0.008
Age	1.82 (1.34–2.48)	<0.001	1.59 (1.16–2.18)	0.004	1.53 (1.11–2.13)	0.010
CCI			1.23 (1.04–1.45)	0.013	1.25 (1.06–1.48)	0.009
WBC count					1.07 (0.97–1.17)	0.177

†Model 1: adjusted for age; ‡model 2: adjusted for model 1 plus CCI; §model 3: adjusted for model 2 plus WBC count.

aHR, adjusted hazard ratio; *CI*, confidence interval; *CCI*, Charlson Comorbidity Index; *WBC*, white blood cell.

Supplementary Table S2. Associated factors for serious complications in the multivariate logistic regression analysis classified by doses of ACE-I or ARB

Variables	ARDS		AKI	
	Adjusted OR (95% CI)	*P* value	Adjusted OR (95% CI)	*P* value
ACE-I/ARB medication				
None	Reference		Reference	
Low-dose	1.55 (0.50–4.76)	0.446	2.38 (0.76–7.46)	0.135
High-dose	6.80 (1.51–30.70)	0.013	2.60 (1.08–6.28)	0.034
Age	1.34 (0.93–1.93)	0.531	1.04 (0.70–1.54)	0.844
CCI	1.11 (0.88–1.40)	0.382	1.15 (0.91–1.45)	0.235
WBC count	1.17 (1.03–1.33)	0.018	1.05 (0.91–1.20)	0.520

ARDS, acute respiratory distress syndrome; *AKI*, acute kidney injury; *CI*, confidence interval; *CCI*, Charlson Comorbidity Index; *OR*, odds ratio; *WBC*, white blood cell.
